# Effectiveness of Microneedling Technique Using Olive Oil on the Severity of Gingival Inflammation and Plaque Accumulation: A Randomised Controlled Trial

**DOI:** 10.7759/cureus.59415

**Published:** 2024-04-30

**Authors:** Swarna Meenakshi P, Subasree S

**Affiliations:** 1 Periodontics, Saveetha Dental College and Hospitals, Saveetha Institute of Medical and Technical Sciences, Saveetha University, Chennai, IND

**Keywords:** subgingival scaling, gingivitis, plaque, olive oil, microneedling

## Abstract

Background:Gingival inflammation, a hallmark of periodontal diseases, serves as a critical focus in oral health research. Characterized by redness, swelling, and bleeding of the gingival tissues, it reflects the body's response to bacterial biofilms accumulating on the tooth surfaces. This inflammatory process, initiated by the interaction between oral bacteria and the host immune system, can lead to a spectrum of periodontal conditions ranging from mild gingivitis to severe periodontitis. Understanding the efficacy of various methods to treat gingival inflammation is essential for refining treatment strategies and enhancing patient satisfaction in the realm of gingival inflammation.

Aim:The objective of the study was to evaluate the efficacy of employing the microneedling technique with olive oil on gingival inflammation and plaque accumulation in individuals with gingivitis.

Materials and methods:Twenty-four individuals diagnosed with plaque-induced gingivitis were selected from Saveetha Dental College, Chennai. Participants were randomly assigned to one of two groups: Group A, comprising 12 individuals who received mechanical periodontal treatment only and Group B, consisting of 12 individuals treated with dermapen and topical olive oil. This involved the creation of microholes in the gingival tissue to enhance the concentration and penetration of the oils through the gingival tissues. Post-intervention assessments of gingival and plaque status were conducted using a gingival index and a plaque index at baseline, one, two, and four weeks. Statistical analysis was done using IBM SPSS Statistics for Windows, version 23 (IBM Corp., Armonk, NY, USA). Intergroup analysis was done using Mann-Whitney test and intra-group analysis was done using Kruskal-Wallis test for all the study parameters. Statistical significance was set at a p-value of less than 0.05.

Results:The mean plaque index scores were 2.02 ± 0.12 and 2.29 ± 0.21 in the subgingival scaling and microneedling with olive oil group respectively in baseline. The scores were 1.83 ± 0.29 and 0.57 ± 0.16 in the subgingival scaling and microneedling with olive oil group respectively at the end of four weeks. The results of plaque index scores were statistically significant between the control and the intervened groups at the end of four weeks with a p value of 0.01*. The mean gingival index scores were 2.09 ± 0.16 and 2.37 ± 0.17 in the subgingival scaling and microneedling with olive oil group in the baseline respectively. The scores were 1.88 ± 0.23 and 0.96 ± 0.21 in the subgingival scaling and microneedling with olive oil group respectively at the end of four weeks. The results of gingival index scores were statistically significant between the control and the intervened groups at the end of four weeks with a p value of 0.01*.

Conclusion:Our research showcased a novel and effective technique, unveiling a significant enhancement in gingival health accompanied by a reduction in both the average gingival index and plaque index*. *

## Introduction

Gingivitis is multifactorial with primary etiology being the local accumulation of microbial pathogens. Additionally, numerous factors like cultural, social, occupational, as well as intra- and interindividual host factors contribute to gingivitis [1]. Plaque-induced gingivitis arises from the presence of bacterial plaque, triggering the body's immune response [2]. The immune response commences by enlisting neutrophils and monocytes to the diseased site, facilitated by cascades of the complement system. Consequently, this immune response can result in the deterioration of gingival tissues, potentially advancing to the destruction of the periodontal attachment apparatus. 

Gingivitis clinically manifests as inflamed, reddened gingival tissues that easily bleed upon probing or brushing. Patients commonly experience gum tenderness, bleeding gums, and swelling, accompanied by a potential increase in gingival crevicular fluid [3]. While the condition is generally reversible with proper oral hygiene measures, untreated gingivitis may progress to more severe periodontal diseases. Monitoring these clinical features is crucial for timely intervention and the prevention of adverse oral health outcomes. Without intervention, untreated cases of gingivitis can lead to increased collagen loss, causing apical migration of the junctional epithelium and subsequent bone loss [4]. Nonetheless, the primary approach to gingivitis treatment involves debridement, encompassing mechanical therapy and the elimination of local factors [5].

The term "mechanical therapy" encompasses both supragingival and subgingival scaling, along with root planing procedures. In conjunction with scaling and root planing for gingivitis, adjunctive therapies play a pivotal role in enhancing treatment outcomes [6]. Antimicrobial agents, such as locally applied antibiotics or antiseptic rinses, effectively target residual bacteria, minimizing the risk of infection recurrence. Furthermore, host modulation agents may be employed to modulate the host response and promote tissue healing [7]. These adjuncts, when integrated into the treatment plan, contribute synergistically to achieving comprehensive management of gingivitis. 

Recently, microneedling has emerged as one of the minimally invasive techniques that have gained attention in the field of dermatology to amplify skin rejuvenation and enhance scar texture [8]. The technique involves the use of microneedles to puncture the surface epithelium, creating fleeting micropores [9]. This process enhances the absorption of topical agents through the stratum corneum, facilitating their efficacy in targeted skin rejuvenation and scar improvement [10]. Furthermore, it induces minute disruptions in the vascular network just beneath the surface epithelium, triggering the innate wound-healing process. 

Oil pulling (OP) refers to a traditional Ayurvedic practice involving mouth rinsing with oil [11]. Historically, this household remedy was believed to effectively address various dental issues and enhance oral hygiene through regular use. OP exhibits positive impacts on oral health without notable adverse reactions like lingering, unpleasant taste, providing a convenient and cost-effective solution [12]. It contributes to a reduction in plaque accumulation, bleeding gums, halitosis, and xerostomia. 

Numerous studies have explored the efficacy of oil pulling using sunflower oil, sesame oil, and coconut oil in alleviating plaque-induced gingivitis [13,14]. One such edible oil is olive oil which has a high content of polyphenolic compounds, particularly tocopherols and bio-phenols, as well as aromatic compounds and antioxidants [15]. The phenolic constituents exert a protective effect by retarding the oxidation of fatty acids, thus inhibiting the onset of rancidity in the oil. Olive oil delays lipid peroxidation and thereby minimizes the free radicals injury to the gingival tissues. Therefore in the present study, an innovative method of microneedling with olive oil was performed for treatment of plaque-induced gingivitis, which was assessed using gingival and plaque index. To the author’s best knowledge, this is the first study to be done using the microneedling technique with olive oil for plaque-induced gingivitis.

## Materials and methods

Study population

The study was performed at the Department of Periodontics, Saveetha Dental College, Chennai, Tamil Nadu, India. The ethical approval was received from the Institutional Ethical Committee at Saveetha Dental College with number of IHEC/SDC/PERIO-2102/23/302. The study was registered in clinical trials registry of India with a number of CTRI/2024/04/065477. Our investigation constituted a randomized comparative interventional study, targeting 24 patients diagnosed with plaque-induced gingivitis. Random allocation was employed to assign participants to two groups. The sample size was estimated with the data using G power 3.0 software, with a power set at 80%. Group A, constituting the control group, comprised 12 individuals who were subjected to subgingival scaling and were advised to follow regular oral hygiene measures as adjunct to scaling. Meanwhile, Group B, designated as the study group, consisted of 12 participants who underwent topical application of organic olive oil using a dermapen. The patients who participated in the study signed a comprehensive consent before the procedure. The consent form explained the treatment protocol and the expected outcomes and adverse effects. 

Inclusion and exclusion criteria

**Figure 1:**
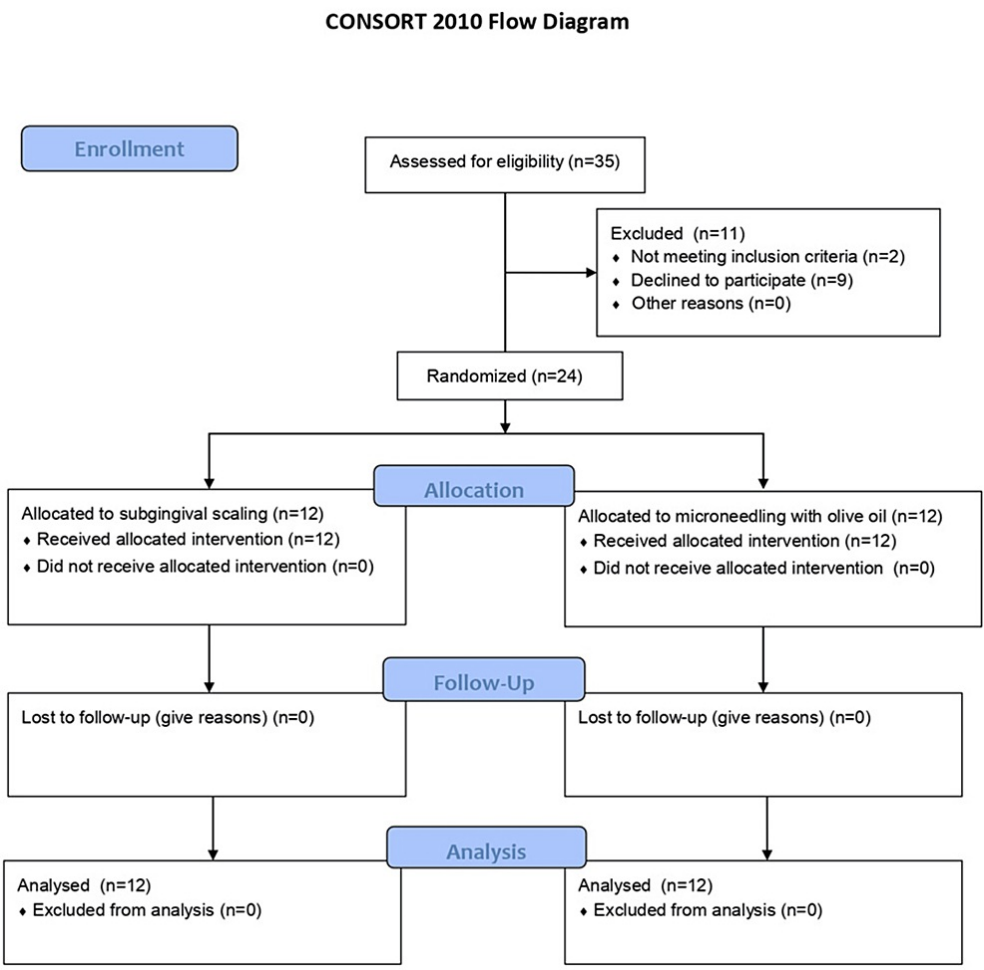
Consort flowchart for patient selection

The inclusion criteria were as follows: 1) patients aged between 20-45 years of age; 2) systemically healthy patients; 3) mild to moderate plaque-induced gingivitis patients; The exclusion factors were: 1) individuals with periodontitis; 2) individuals who smoke or those who have tobacco related habits; 3) partially edentulous individuals; 4) individuals who are diagnosed with drug-induced gingival enlargement; 5) third molars were excluded (Figure 1).

Clinical Protocols

Subgingival Scaling

**Figure 2:**
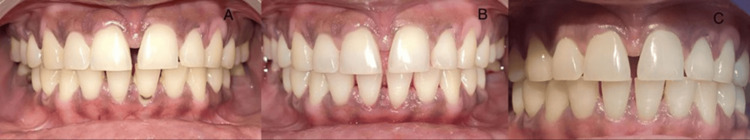
Subgingival scaling a) Preoperative picture; b) Immediate postoperative picture; c) Four-week review picture

All subjects underwent subgingival scaling employing a Piezoelectric ultrasonic scaler to eliminate local factors. The piezoelectric scaler employs high-frequency vibrations generated by a piezoelectric crystal, facilitating precise and controlled scaling. The piezoelectric scaler operates with ultrasonic vibrations that break down and dislodge deposits on teeth without causing significant trauma to surrounding tissues. Preceding the treatment, participants received oral hygiene instructions, encompassing proper tooth brushing techniques and exclusive reliance on flossing (Figure 2).

Microneedling With Olive Oil

**Figure 3:**
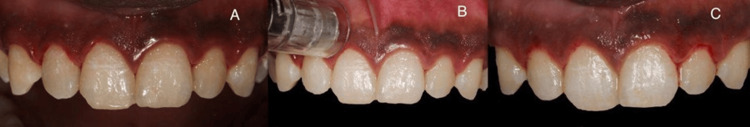
Microneedling technique a) Preoperative picture; b) Intraoperative picture with dermapen; c) Immediate postoperative picture

Microneedling of the mucosa was performed using dermapen needles, employing an intermittent back-and-forth motion in the anterior maxillary and mandibular region for 30-40 seconds, reaching a depth of 1.5 mm. The dermapen (Electric Auto Stamp Dermapen; Dr. Pen, Perth, Australia) consisted of a handle, disposable heads and guides to alter the height of the needle and momentum. The device allows for adjustable penetration depth, ranging from 0.2 mm to 3 mm, based on the gingival thickness, and this depth can be set using the device's penetration depth settings. The needle tip comprises 12 to 24 needles arranged in rows, providing six speed modes that span from 412 cycles/min at the lowest speed to 700 cycles/min at the highest speed [16]. Application of olive oil followed the microneedling, where cotton rolls were used to apply oil to the upper anterior gingiva and lower anterior gingiva in a circular motion when uniform bleeding pinpoints were observed. The cotton was placed on the gingiva for five minutes. Patients were given instructions to avoid acidic or hot beverages for 24 hours and refrain from tooth brushing for a day to prevent any mechanical trauma to the treated gingiva (Figure 3).

**Figure 4:**
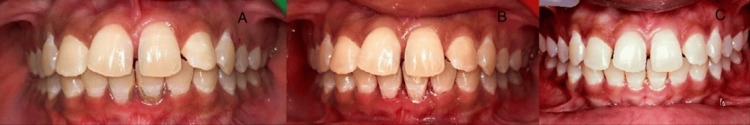
Microneedling technique a) Preoperative picture; b) Immediate postoperative picture; c) Four-week review picture

Each patient was re-evaluated at four point intervals: baseline, one week, two weeks and one month after treatment (Figure 4).

Clinical assessment

Gingival Index

Clinical evaluation was done by engaging William’s probe approximately 1 to 2 mm to the margin of the gingiva at an angulation of 45 degrees with minimal axial pressure and scouring from one proximal surface to another along the labial and lingual aspects of the tooth. Gingivitis was evaluated on six surfaces per tooth (buccal, lingual, mesial, mesiobuccal, distal, distobuccal) on all permanent teeth, except third molars. Gingival index (GI) was calibrated with a score of 0 to 3 and then the sites were summed up and divided by six to calculate the GI of individual teeth [17].

A score of 0 indicates the absence of inflammation. A score of 1 signifies the presence of mild inflammation, and color changes, with no observed bleeding. A score of 2 indicates moderate inflammation, characterized by redness and bleeding upon probing. A score of 3 denotes severe inflammation, marked by pronounced redness, edema, and spontaneous bleeding. 

The gingival index of each tooth was evaluated, and then all final scores were summed up together and divided by six to assess the average GI with the following calibrations and interpreted as follows: Scores ranging from 0.1 to 1 indicated mild gingivitis, while scores falling between 1.1 and 2 were indicative of moderate gingivitis. Severe gingivitis was characterized by scores within the range of 2.2 to 3. 

Plaque Index

In assessing plaque accumulations on the teeth, the plaque index (PI) was employed as a quantitative measure. A systematic scoring system was applied to each of the teeth's four surfaces (buccal, lingual, mesial, and distal) resulting in scores ranging from 0 to 3. The cumulative scores for all surfaces of a given tooth were then summed and divided by four, yielding a precise PI value for each tooth [18].

A score of 0 indicates the absence of plaque. A score of 1 denoted plaque adhering to gingival margin and adjacent tooth areas, visible only upon using a probe on the tooth surface and cannot be seen with naked eye. A score of 2 represented a moderate accumulation of soft deposits within the gingival pocket, observable without the aid of a probe. A score of 3 indicated an abundance of soft deposits within the gingival pocket and/or on the tooth and gingival margin, covering interdental areas.

The PI for individual teeth was assessed, and then all final scores were added together and divided by four to get average PI and was interpreted as follows: A score of 0 signified excellent oral hygiene, while scores ranging from 0.1 to 1 were indicative of good oral hygiene. Oral hygiene was considered fair for scores falling within the range of 1.1 to 2, and a score between 2.1 and 3 was associated with bad oral hygiene. 

Statistical analysis

The mean and standard deviations of the scores were evaluated. For gingival index, Kruskal-Wallis test was used to compare between different time intervals (baseline, one week, two weeks and four weeks) in the same group followed by Mann-Whitney test to evaluate the difference between the groups. To assess the plaque index scores, Mann-Whitney test was used to evaluate the difference between groups and Kruskal-Wallis test was utilized to assess the difference between intervals (baseline, one week, two weeks and four weeks) within the same group. Statistical analysis was done using Statistical Package for Social Sciences (SPSS) software version 23 (IBM Corp., Armonk, NY, USA). A p-value less than 0.05 was considered to be statistically significant.

## Results

The study included 24 patients who were diagnosed with gingivitis. They were segregated into two groups, with 12 patients in each group. Fifty percent were men (n=12) and 50% were women (n=12). 

Gingival index scores

The mean gingival index scores were 2.09 ± 0.16 and 2.37 ± 0.17 in the subgingival scaling and microneedling with olive oil group respectively at baseline. This indicated most of the patients had severe gingivitis in the baseline. The gingival index scores were 1.74 ± 0.38 and 1.70 ± 0.31 in the scaling and microneedling with olive oil group respectively post one week. The scores were 1.88 ± 0.23 and 0.96 ± 0.21 in the subgingival scaling and microneedling with olive oil group respectively at the end of four weeks. The results of the current study revealed that the gingival index scores were statistically significant between the control and the intervened groups at the end of four weeks with a p-value of 0.01* (Table 1).

**Table 1:** Depicts the mean and standard deviation of gingival index scores between the studied groups Statistically significant difference in gingival index scores were observed between the scaling group and microneedling group at the end of four weeks with a p-value of 0.01*. Also statistically significant difference was seen within the microneedling group between baseline and four weeks (p<0.01*).

Study groups	Gingival index (Subgingival Scaling Group)		Gingival index (Microneedling Group)		p-value
	Mean±SD		Mean±SD		
Baseline	2.09±0.16		2.37±0.17		0.66
1 week	1.74±0.38		1.70±0.31		0.72
2 weeks	1.70±0.39		1.59±0.17		0.63
4 weeks	1.88±0.23		0.96±0.21		0.01*
p-value (Baseline vs 4 weeks)	0.45		<0.01*		

Plaque index scores

The mean plaque index scores were 2.02 ± 0.12 and 2.29 ± 0.21 in the scaling and microneedling with olive oil group respectively. This indicated most of the patients had severe gingivitis in the baseline. The plaque index scores were 1.84 ± 0.18 and 1.75 ± 0.17 in the scaling and microneedling with olive oil group respectively post one week. There was no statistically significant difference in the plaque index scores at the end of the first week between the groups. The scores were 1.83 ± 0.29 and 0.57 ± 0.16 in the scaling and microneedling and olive oil group respectively at the end of four weeks. Therefore, the plaque index scores were statistically significant between the control and the intervened groups at the end of four weeks with a p-value of 0.01* (Table 2). 

**Table 2:** Depicts mean and standard deviation of plaque index scores between the studied groups Statistically significant difference in plaque index scores were seen between scaling group and microneedling group at the end of four weeks with a p-value of 0.01*. Also statistically significant difference was observed within the microneedling group between baseline and four weeks (p<0.01*).

Study groups	Plaque index (Subgingival Scaling Group)		Plaque index (Microneedling Group)		p-value
	Mean± SD		Mean± SD		
Baseline	2.02± 0.12		2.29± 0.21		0.54
1 week	1.84± 0.18		1.75± 0.17		0.61
2 weeks	1.70± 0.34		1.64± 0.24		0.56
4 weeks	1.83± 0.29		0.57± 0.16		0.01*
p-value (Baseline vs 4 weeks)	0.52		<0.01*		

## Discussion

In the current study, all the participants of the test group and the non-intervention group received mechanical therapy with subgingival scalers, and all participants were dictated on the optimal oral hygiene instructions prior to treatment. Additionally, we utilized a microneedling pen to usher organic oils into the gingival crevice for the experimental group. Unfortunately, due to the straight design of the Dermapen's head (microneedling pen), its use in the posterior areas was limited. Despite this limitation, dermapen allows sustained delivery of therapeutic agents promoting anti-inflammatory responses and aids in reducing microbial load [19]. Additionally, the controlled micro-injuries induced by the microneedling process stimulate collagen production, promoting tissue regeneration and overall oral health.

Additionally, in the current research we used natural oil pulling as an alternative strategy for chemical mouthwashes. Phenols, commonly found in antiseptic mouthwashes, exhibit broad-spectrum antimicrobial activity. While effective in reducing oral bacteria, prolonged and frequent use of phenol-containing mouthwashes may lead to undesirable side effects. Phenols can cause mucosal irritation and a burning sensation in the mouth, potentially impacting patient compliance [20]. Stannous fluoride, another common ingredient in mouthwashes, is known for its antiplaque and anticaries properties. Stannous fluoride has been linked to tooth staining, commonly referred to as "stannous staining" or "stannous tattoos." This aesthetic concern can be a significant drawback for individuals seeking both oral health benefits and a visually pleasing smile. Staining may occur due to the formation of insoluble complexes on tooth surfaces, leading to discoloration over time [21]. On the other hand, natural oils like olive oil are easily feasible, and have exhibited many positive impacts on oral health with limited adverse reactions [22].

The study's findings revealed a statistically significant reduction in GI scores in the experimental group compared to the non-intervention group. Moreover, a substantial reduction in GI scores indicated a mitigated severity of gingival inflammation. The results showed that GI scores exhibited reduction in values from the baseline to four weeks in the experimental group which was statistically significant, while the subgingival scaling group also presented a reduction in scores when compared to baseline values which was not statistically significant. This result was in accordance with previous research conducted by Mostafa et al. [23]. The observed decrease in gingival inflammation in microneedling with olive oil group can be attributed to the antioxidant effects of oil-pulling. This process activates salivary enzymes and facilitates the removal of toxins from the bloodstream [24]. This chemical reaction reduces the adhesion of plaque and alleviates gingival inflammation. The combination of these mechanisms underscores the potential efficacy of oil-pulling as a preventive measure against gingival inflammation.

The outcomes of the PI demonstrated a decrease in scores from the initial assessment to the fourth week between the experimental and control groups exhibiting a significant difference. The decline in PI scores within the experimental group could potentially be attributed to the interaction between the oleic acid present in olive oil and sodium hydroxide in saliva [25]. This interaction is believed to be responsible for the detoxification process, leading to a decrease in deposition of plaque. These results were in accordance with Zumbo et al. who revealed that there was a statistically significant reduction in the scores of plaque index and bleeding index when using olive oil [26]. The reduction in plaque index scores in the microneedling with olive oil group may be in accordance with the findings of Filomena et al. who imparted that olive oil has antimicrobial activity against gram-negative and gram-positive microorganisms that contribute to plaque accumulation and microbial aggregation [27]. This infers that olive oil has a significant impact on plaque reduction.

Furthermore, Zakrzewski et al. 2020 concluded that olive oil has antibacterial activity against S. mutans and Candida albicans [28]. These findings confirm the antimicrobial efficacy of olive oil. These findings were also concluded by Maria et al. 2020 who reported that olive oil showed a significant reduction in antimicrobial activity against S. mutans when compared to commercial chlorhexidine mouthwash [29]. While the decrease in plaque index scores in scaling group was only related to anti-infective mechanical therapy, where the main local factors like plaque and calculus that cause the gingival inflammation were removed.

Improvement in gingival health is attributed to the antioxidant properties of organic acid and the regenerative effect of the microneedling technique. Microneedling is thought to influence the process of neovascularization and neocollagenesis which is initiated through the migration and proliferation of fibroblasts, along with the deposition of the intercellular matrix. This sequence of events contributes to the mucosa acquiring a tightened appearance [30]. This dual action underscores the multifaceted approach contributing to the observed improvement in gingival health. However, it is crucial to acknowledge that the study's sample size was limited to these selected patient groups. Therefore, the limitation of the current study is that in order to validate and generalize these findings, further future studies with larger sample sizes are needed.

## Conclusions

The present study showcased an innovative and effective technique, demonstrating a significant enhancement in gingival health and a decrease in both average GI and PI compared to sole mechanical debridement. Notably, the application of olive oil using microneedling exhibited a superior improvement in reduction of gingival inflammation compared to mechanical debridement alone. Therefore, employing olive oil pulling therapy through microneedling could be advocated as a viable treatment approach for periodontal diseases, particularly those that prove refractory to conventional interventions.
